# Monozentrische Kohortenanalyse des anästhesiologischen Managements von Schwangeren mit angeborenen Herzfehlern

**DOI:** 10.1007/s00101-026-01690-2

**Published:** 2026-05-07

**Authors:** Emmanuel Schneck, Melanie Markmann, Annahita Bahadori, Emmanuella Iyoha, Frank Oehmke, Roland Axt-Fliedner, Aline Wolter, Monika Lüdemann, Thomas S. Zajonz, Matthias Müller

**Affiliations:** 1 Klinik für Anästhesiologie, operative Intensivmedizin und Schmerztherapie, Universitätsklinikum Gießen und Marburg (UKGM), Justus-Liebig-Universität Gießen, Rudolf-Buchheim-Straße 7, 35392 Gießen, Deutschland; 2 Klinik für Anästhesiologie, operative Intensivmedizin, Vinzenz-Krankenhaus, Hannover, Deutschland; 3 Klinik für Gynäkologie und Geburtshilfe, Universitätsklinikum Gießen und Marburg (UKGM), Justus-Liebig-Universität Gießen, Gießen, Deutschland; 4 Zentrum für pränatale Medizin und fetale Therapie, Universitätsklinikum Gießen und Marburg (UKGM), Justus-Liebig-Universität Gießen, Gießen, Deutschland; 5 Klinik für Kinderkardiologie, Zentrum für Erwachsene mit angeborenen Herzfehlern, Universitätsklinikum Gießen und Marburg (UKGM), Justus-Liebig-Universität Gießen, Gießen, Deutschland

**Keywords:** Anästhesie, Hämodynamik, Sectio caesarea, Angeborene Herzfehler, Anesthesia, Hemodynamics, Cesarean section, Congenital heart disease

## Abstract

**Einleitung:**

Mit der steigenden Zahl Erwachsener mit angeborenen Herzfehlern (EMAH) in Deutschland nimmt auch die Zahl schwangerer EMAH-Patientinnen zu, was anästhesiologische und geburtshilfliche Teams zunehmend vor komplexe Herausforderungen stellt. Diese Studie untersucht anästhesiologische Managementstrategien und postoperative Versorgungsanforderungen bei EMAH-Patientinnen, die in einem tertiären Versorgungszentrum behandelt wurden.

**Methoden:**

Diese retrospektive monozentrische Studie umfasste EMAH-Patientinnen, die zwischen 2004 und 2023 eine Anästhesie für eine Entbindung, einen Kaiserschnitt oder eine geburtshilfliche Operation erhielten. Es erfolgte ein 1:2-Matching auf ausgewählte klinische Basisvariablen mit geburtshilflichen Patientinnen ohne angeborenen Herzfehler zur deskriptiven Einordnung.

**Ergebnisse:**

Insgesamt wurden 391 Fälle in die Studie eingeschlossen, davon 131 EMAH-Fälle und 260 Fälle der Vergleichsgruppe (VG) (mWHO I: 44,2 %, mWHO II: 23,8 %, mWHO II-III: 20,5 %, mWHO III und IV: 11,5 %). Spinal- und Epiduralanästhesien wurden signifikant häufiger bei Patientinnen mit niedrigeren mWHO-Klassen eingesetzt (*p* = 0,017). Alle mWHO IV-Patientinnen benötigten eine Allgemeinanästhesie durch Anästhesisten mit Erfahrung in der Kardioanästhesie. Postoperativ wurden EMAH-Patientinnen signifikant häufiger intensivmedizinisch betreut (ITS: 6,6 % vs. 1,6 %, *p* = 0,02; IMC: 13,9 % vs. 3,7 %, *p* < 0,001). Insgesamt war die anästhesiologische Komplikationsrate niedrig und mit der VG vergleichbar.

**Diskussion:**

Eine geburtshilfliche Anästhesie mit niedrigen mütterlichen Komplikationsraten war auch bei EMAH-Patientinnen mit hoher Erkrankungsschwere möglich. Alle Anästhesieverfahren erwiesen sich als durchführbar, wenn sie an das Risikoprofil angepasst wurden.

**Graphic abstract:**

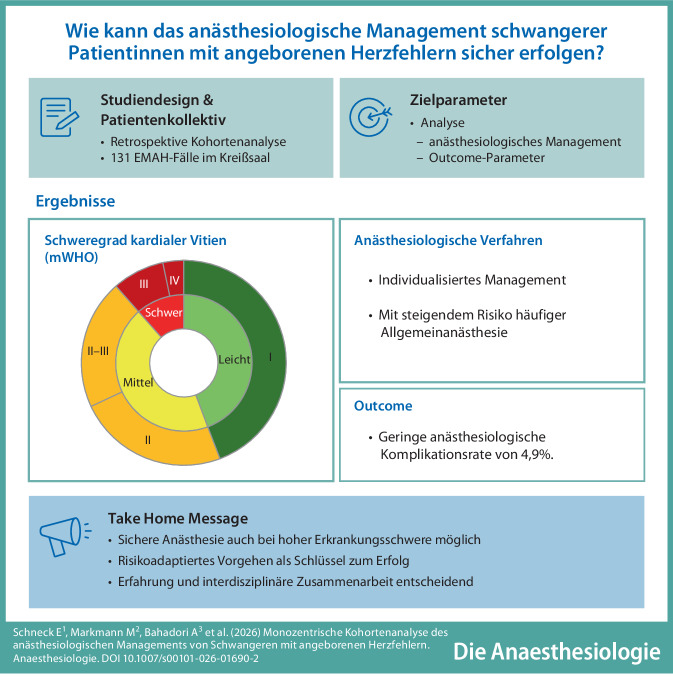

## Abstract (en)

**Introduction:**

The number of pregnant women with adult congenital heart disease (ACHD) is increasing, posing growing challenges for obstetric anaesthesia. This study analysed anaesthetic strategies and postoperative care in ACHD patients treated at a tertiary care centre.

**Methods:**

This retrospective single-centre study included ACHD patients who received anaesthesia for delivery, caesarean section or obstetric surgery between 2004 and 2023. A comparison group of obstetric patients without congenital heart disease was selected based on baseline characteristics.

**Results:**

A total of 391 cases were included (131 ACHD, 260 comparison group). Most patients were classified as mWHO I (44.2%) or II (23.8%), with 20.5% mWHO II–III and 11.5% mWHO III and IV. Spinal and epidural anaesthesia were used more frequently in lower mWHO classes (*p* = 0.017). All mWHO IV patients required general anaesthesia provided by ACHD-experienced anaesthetists. ACHD patients more frequently required postoperative ICU or IMCU care (6.6% vs. 1.6%, *p* = 0.02; 13.9% vs. 3.7%, *p* < 0.001). Anaesthesiologic complication rates were low in both groups.

**Conclusion:**

Obstetric anaesthesia was feasible with low complication rates, even in severe ACHD, when management was individualised and interdisciplinary. All anaesthetic procedures proved to be feasible when adapted to the risk profile.

## Einleitung

Fortschritte in Diagnostik und Therapie haben zu einer stetig wachsenden Population von Erwachsenen mit angeborenen Herzfehlern (EMAH) geführt. Allein in Deutschland sind etwa 300.000 Menschen betroffen, welche unkorrigierte, korrigierte (mit Residualbefunden) oder palliativ behandelte Herzfehler aufweisen können. Dabei erreichen mit steigenden Überlebensraten und Lebensqualität immer mehr EMAH-Patientinnen das reproduktive Alter und entscheiden sich für eine Schwangerschaft [6, 22]. Dies stellt die interdisziplinären geburtshilflichen und anästhesiologischen Teams vor erhebliche Herausforderungen, da die Komplexität und Heterogenität angeborener Herzfehler sowohl die mütterliche als auch die fetale Morbidität und Mortalität beeinflussen können. Komplikationen treten bei etwa 10 % der Schwangerschaften von EMAH-Patientinnen auf, und kardiovaskuläre Komplikationen bleiben eine führende und häufig vermeidbare Ursache für eine erhöhte mütterliche Mortalität in dieser Gruppe [1, 29].

Obwohl europäische und amerikanische Leitlinien allgemeine Empfehlungen für das Management schwangerer Patientinnen mit kardiovaskulären Erkrankungen bieten, fehlen spezifische Richtlinien für die geburtshilfliche und anästhesiologische Versorgung von EMAH-Patientinnen [1, 27, 29]. Eine aktuelle Übersichtsarbeit der *American Heart Association* (AHA) zu Anästhesieverfahren bei Erwachsenen mit Herzerkrankungen während der Schwangerschaft skizziert zwar allgemeine Prinzipien, geht jedoch nicht auf die besonderen Bedürfnisse von Patientinnen mit angeborenen Herzfehlern ein [15]. Evidenzbasierte Empfehlungen für die Auswahl geeigneter Anästhesieverfahren, die auf individuelle Herzfehler zugeschnitten sind, sind rar. Die aktuellen Strategien basieren oft auf Fallberichten, kleinen retrospektiven Studien und klinischer Erfahrung aus anderen Bereichen der EMAH-Versorgung. Auch die Empfehlungen der AHA hinsichtlich der anästhesiologischen Versorgung von Kindern mit angeborenen Herzfehlern bei nicht-herzchirurgischen Eingriffen können nur eingeschränkt auf schwangere EMAH-Patientinnen übertragen werden [18]. Angesichts der seltenen Prävalenz von Schwangerschaften in dieser Patientengruppe werden in der klinischen Praxis aufgrund mangelnder systematischer Evidenz häufig individualisierte, risikobasierte Managementstrategien angewendet.

Das Gießener Kinderherzzentrum hat als zertifiziertes EMAH-Zentrum in den letzten 20 Jahren eine wachsende Zahl schwangerer EMAH-Patientinnen mit individualisierten Versorgungsplänen betreut. In einer Ära begrenzter personeller Ressourcen priorisiert unser Zentrum Hochrisikofälle, um eine hoch spezialisierte Versorgung dort zu ermöglichen, wo sie am dringendsten benötigt wird [19]. Bisher erfolgte keine systematische Auswertung der anästhesiologischen Verfahren. Diese retrospektive Studie zielt daher darauf ab, das anästhesiologische Management schwangerer EMAH-Patientinnen zu analysieren, mit besonderem Fokus auf die Identifizierung von Risikoprofilen, die mit einem ungünstigen mütterlichen Outcome assoziiert sind.

## Methoden

### Studiendesign

Diese retrospektive, monozentrische Studie wurde von der Ethikkommission der Justus-Liebig-Universität Gießen genehmigt (AZ 117/20) und folgte den Prinzipien der Deklaration von Helsinki. Die Berichterstattung erfolgte gemäß den STROBE-Richtlinien [7]. Es wurden Daten aller EMAH-Patientinnen ≥ 18 Jahre erfasst, die zwischen dem 1. März 2004 und dem 31. März 2023 an der Universitätsklinik Gießen eine Anästhesie für eine Entbindung, einen Kaiserschnitt oder eine geburtshilfliche Operation erhielten.

### Datenerfassung und Studienparameter

Patientinnen wurden manuell durch Screening der geburtshilflichen Akten und des Anästhesie-Dokumentationssystems (NarkoData^®^, IMESO, Deutschland) identifiziert.

Das 1:2-Matching erfolgte auf ausgewählte klinische Basisvariablen, einschließlich Alter, schwangerschaftsbezogener Erkrankungen (z. B. Präeklampsie, schwangerschaftsinduzierte Hypertonie, Diabetes) sowie des BMI (maximale Abweichung 10 %). Angaben zum BMI basierten auf der Dokumentation aus Geburtshilfe und Pränataldiagnostik. Erfasst wurde jeweils das erste dokumentierte Gewicht. Das Gewicht zu Beginn der Schwangerschaft wurde aus dem Mutterpass im Rahmen der Pränataldiagnostik übernommen, das Gewicht am Ende der Schwangerschaft aus der geburtshilflichen Dokumentation zum Zeitpunkt der Entbindung. Die Körpergröße wurde einmalig erfasst, da von keiner relevanten Veränderung im Verlauf der Schwangerschaft auszugehen ist. Der BMI wurde entsprechend für die jeweiligen Zeitpunkte berechnet. Das Matching diente dabei der deskriptiven Einordnung der EMAH-Gruppe und nicht der Ableitung kausaler Effekte. Da die klinische Dokumentation bei vaginalen Entbindungen im Vergleich zur Dokumentation bei Sectio caesarea über den langen Beobachtungszeitraum deutlich weniger detailliert war, wurden in die Vergleichsgruppe (VG) nur Patientinnen, welche eine Sectio caesarea erhielten, eingeschlossen. Daher wurde die Art der Entbindung beim Matching nicht berücksichtigt.

Der primäre Endpunkt war die Beschreibung des anästhesiologischen Managements der Studienpopulation über 20 Jahre. Hierfür wurden neben den pseudonymisierten Patientencharakteristika, die Art des Herzfehlers, das perioperative Management und die Erfahrung des behandelnden Anästhesisten erhoben. Die Schwere des Herzfehlers wurde nach den Leitlinien der *European Society of Cardiology* (ESC) und der AHA (mWHO-Klassifikation) eingestuft. Letztere empfiehlt die Klassifikation angeborener Herzfehler nach dem *American College of Surgeons National Surgical Quality Improvement Program* (ACS-NSQIP) unter Berücksichtigung der verbliebenen Läsionslast und des funktionellen Status [8, 18, 21, 27]. Da dieser Score nur für Kinder validiert wurde, passten wir ihn für schwangere Patientinnen an: mWHO I wurde als ACS-NSQIP „*minor congenital heart disease*“, mWHO II und mWHO II–III als „*major congenital heart disease*“ und mWHO III sowie IV als „*severe congenital heart disease*“ klassifiziert.

Der sekundäre Endpunkt war das Auftreten von unerwünschten Ereignissen während oder nach der Entbindung im Vergleich zur Vergleichsgruppe. Unerwünschte Ereignisse wurden definiert als geburtshilfliche Komplikationen während der Entbindung (z. B. Geburtsstillstand), pathologische fetale Überwachung, übermäßige Blutung (≥ 500 ml bei vaginaler Geburt oder ≥ 1000 ml bei Sectio), Uterusruptur, Arrhythmien (außer Sinusarrhythmie), Hypoxie (Beatmungspflicht) oder schwere Hypotonie mit Notwendigkeit einer Katecholamintherapie (Adrenalin, Noradrenalin, Milrinon oder Dobutamin, jedoch nicht Theodrenalin/Cafedrin).

Der Schwerpunkt dieser retrospektiven Studie lag auf perioperativen Ereignissen vom Zeitpunkt der Entbindung bis zur Entlassung aus dem Aufwachraum, der Intermediate Care (IMC) oder der Intensivstation (ITS). Langzeitverlaufsdaten über diesen Zeitraum hinaus lagen nicht konsistent vor und wurden daher nicht berücksichtigt. Die Mortalität wurde als innerklinische Sterblichkeit bis zur Entlassung definiert.

### Anästhesiologisches Management

Die Qualifikation des verantwortlichen Anästhesisten sowie die verwendeten Anästhesieverfahren sind im Ergebnisteil detailliert beschrieben. Sofern nicht anders angegeben, bestand die Spinalanästhesie aus 0,025 mg Fentanyl, 1 ml Mepivacain 1 % und Bupivacain 0,5 % (Dosierung: doppelte Körpergröße in cm geteilt durch 100 -0,2 ml).

Die Periduralanästhesie unter der Geburt erfolgte mit 10 µg Sufentanil und 5–8 ml Ropivacain 0,2 %. Für Sectio caesarea bestand die Periduralanästhesie aus 10 µg Sufentanil und 12–15 ml Ropivacain 0,75 %.

Detaillierte Informationen zum Management der Allgemeinanästhesie sind im Ergebnisteil beschrieben.

### Statistische Analyse

Es wurde eine deskriptive statistische Analyse durchgeführt. Kategoriale Variablen werden als absolute und relative Häufigkeiten angegeben, numerische Variablen als Median mit Interquartilsabstand (IQR).

Gruppenunterschiede wurden mit dem Student-t-Test bei Normalverteilung und ansonsten mit dem Mann-Whitney-U-Test oder dem Wilcoxon-Test für kontinuierliche Variablen analysiert. Zusammenhänge zwischen kategorialen Variablen wurden mit dem Fisher-Test geprüft. Ein *p*-Wert ≤ 0,05 wurde als statistisch signifikant gewertet.

Alle statistischen Analysen wurden mit der Statistiksoftware R (Version 4.2.3, Release 15.03.2023; www.r-project.org) durchgeführt.

## Ergebnisse

### Patientencharakteristika

Insgesamt wurden 391 Fälle in die Studie eingeschlossen, darunter 122 EMAH-Patientinnen, von denen acht Patientinnen mehr als einen anästhesiologischen Eingriff aufgrund von geburtshilflichen Interventionen erhielten (insgesamt 131 EMAH-Fälle). Sofern nicht anders angegeben, beziehen sich die Angaben auf die Anzahl der EMAH-Patientinnen (*n* = 122) und nicht auf die Fallanzahl. Die Vergleichsgruppe bestand aus 244 Patientinnen mit insgesamt 260 Fällen.

**Abb. 1:**
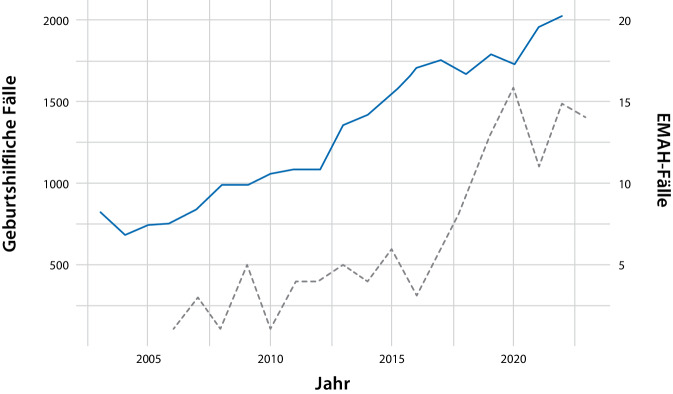
Entwicklung der EMAH-Fälle über den Studienzeitraum. Die *gestrichelte* Linie zeigt die Anzahl der geburtshilflichen EMAH-Fälle, während die *blaue* Linie die Gesamtzahl der Entbindungen darstellt. *EMAH* erwachsene Patientinnen mit angeborenen Herzfehlern

Die schwangerschafts- und geburtsassoziierten Diagnosen wurden gemäß dem definierten Matching-Schema berücksichtigt. Die Basischarakteristika sind in Tab. 1 dargestellt. Im Verlauf des Studienzeitraums stieg sowohl die Gesamtzahl der Entbindungen als auch die Zahl der EMAH-Fälle an (Abb. 1).

**Tab. 1:** Demographische Daten und Basischarakteristika

Parameter (Patienten)	EMAH (*n* = 122)	VG (*n* = 244)	*p*-Wert
*Alter (Jahre)*	29,8 ± 5,1	29,8 ± 5,1	0,97
*BMI zur Erstbestätigung der Schwangerschaft (kg/m*^*2*^*)*	24,2 ± 4,7	23,4 ± 4,6	0,15
*BMI zum Zeitpunkt der Entbindung (kg/m*^*2*^*)*	27,8 ± 4,8	28,0 ± 4,7	0,73
*Dauer der Schwangerschaft (Tage)*	262,5 ± 27,1	261,4 ± 22,3	0,71
*Gravida (n; MW* *±* *SD)*	0,89 ± 1,34	1,1 ± 1,28	0,14
*Para (n; MW* *±* *SD)*	0,54 ± 0,81	0,72 ± 0,91	*0,046*
Nebendiagnosen, n (%)			*0,004*
*Keine*	92 (75,4)	204 (85,2)	0,51
*Arterielle Hypertension*	8 (6,6)	4 (1,6)	*0,02*
*Diabetes mell. (nicht schwangerschaftsassoziiert)*	4 (3,3)	5 (2,0)	0,47
*Chronische Niereninsuffizienz*	1 (0,8)	0	0,32
*Hypothyreose*	21 (17,2)	30 (13,2)	0,15
*Syndromatische Erkrankungen*	3 (2,5)	0	*0,03*
Dauermedikation			
*Oral Antikoagulation vor Schwangerschaft, n (%)*	9 (7,4)	14 (5,7)	0,65
*Thrombozytenaggregationshemmung, n (%)*	16 (13,1)	6 (2,5)	< 0,001
Kardiale Dauermedikation n (%)			
*Keine*	88 (72,1)	0	–
*LMWH in therapeutischer Dosierung*	9 (7,4)	0	
*β‑Blocker*	23 (17,6)	0	
*Aspirin*	12 (9,2)	0	
*Sildenafil*	2 (1,6)	0	
*Flecainid*	1 (0,8)	0	

Statistisch signifikante Unterschiede (*p* < 0,05) sind kursiv markiert. *p*-Werte werden zur deskriptiven Beschreibung von Gruppenunterschieden angegeben. Bei der Interpretation müssen die strukturellen Gruppenunterschiede hinsichtlich des Geburtsmodus (ausschließlich Sectio caesarea in der Vergleichsgruppe) beachtet werden.

*BMI* Body-Mass-Index, EMAH erwachsene Patientinnen mit angeborenen Herzfehlern, *LMWH* niedermolekulares Heparin, *MW* Mittelwert, *SD* Standardabweichung, *VG* Vergleichsgruppe

### Charakterisierung der kardialen Vitien

**Abb. 2:**
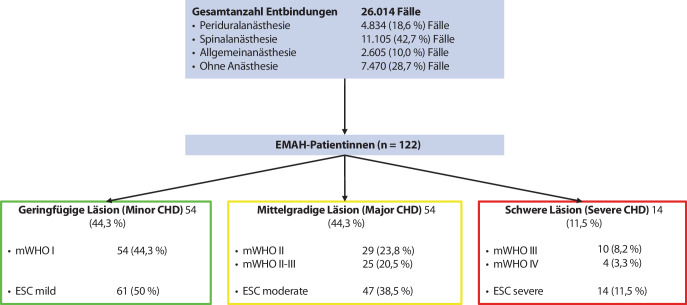
Komplexität der kongenitalen Vitien. Die Definition erfolgte gemäß der *European Society of Cardiology* (mild, moderat, schwer) bzw. der modifizierten WHO-Klassifikation (I–IV) [5, 21]. Die Einteilung in geringgradige, mittelschwere und schwere angeborene Herzfehler erfolgte wie in den Methoden beschrieben und basiert auf dem AHA-Statement zu perioperativen Aspekten nichtkardialer Eingriffe bei pädiatrischen Patientinnen [5]. *CHD* congenital heart disease, *EMAH* Erwachsene Patientinnen mit angeborenen Herzfehlern, *ESC* European Society of Cardiology, *mWHO* Modified World Health Organization Classification

Die zugrunde liegenden Arten der angeborenen Herzfehler sind in Tab. 2 dargestellt. Von allen Patientinnen wurden 68,1 % als milder angeborener Herzfehler (mWHO I und II) klassifiziert. Mit zunehmender Schwere des angeborenen Herzfehlers nahm die Fallhäufigkeit ab (Abb. 2).

**Tab. 2:** Charakterisierung der angeborenen Herzfehler (mehrere Diagnosen pro Patientin möglich)

Parameter	EMAH (*n* = 122)
Art des kongenitalen Vitiums, *n* (%)	
*Korrigierter VSD*	26 (21,3)
*Pulmonalstenose*	25 (20,5)
*Korrigierter ASD*	20 (16,4)
*Kongenitale Aortenstenose*	16 (13,1)
*Kongenitale Mitralstenose*	14 (11,5)
*Korrigierte TGA*	13 (10,7)
*Kongenitale Kardiomyopathie*	13 (10,7)
*Korrigierte Aortenstenose*	13 (10,7)
*Korrigierte TOF*	11 (9,0)
*Kongenitales Trikuspidalklappenvitium*	9 (7,4)
*Rechtsventrikuläre Dysfunktion und pulmonale Hypertension*	6 (4,9)
*Korrigierter DORV*	4 (3,3)
*PFO*	4 (3,3)
*UVH*	3 (2,5)
*Korrigierter AVSD*	2 (1,6)
*Korrigierte Trikuspidalklappenatresie*	2 (1,6)
*Korrigierter PDA*	2 (1,6)
*Korrigierte Pulmonalatresie*	1 (0,8)
*Korrigierte absent pulmonary valve*	1 (0,8)
Zyanose, *n* (%)	
*Azyanotisch*	115 (94,3)
*Zyanotisch*	1 (0,8)
* Fehlende Angaben*	6 (4,9)
Pulmonale Hypertension	5 (4,1)
Fontan-Zirkulation, *n* (%)	
*Hemifontan*	1 (0,8)
*Fontan*	4 (3,3)
Arrhythmie, *n* (%)	
*Supraventrikuläre Tachykardie*	13 (10,7)
*Ventrikuläre Ektopien*	3 (2,5)
*Ventrikuläre Tachykardie*	2 (1,6)
*Schrittmacher/ICD*	8 (6,1)
NYHA zum Beginn der Schwangerschaft, *n* (%)	
*I*	77 (63,1)
*II*	23 (18,9)
*III*	2 (1,6)
*IV*	0 (0)
*Fehlende Angaben*	20 (16,4)
NYHA zum Ende der Schwangerschaft, *n* (%)	
*I*	54 (44,3)
*II*	33 (27,0)
*III*	11 (9,0)
*IV*	0 (0)
Fehlende Angaben	24 (19,7)

Mehrfachantworten waren bei der Art der Diagnose entsprechend der Heterogenität der Herzfehler erlaubt. Bei den Parametern Zyanose, pulmonale Hypertonie, Fontan-Zirkulation, Arrhythmie sowie Schrittmacher/ICD wurden nur die Patientinnen angegeben, die diese Parameter auch erfüllt hatten, da für diese Parameter vollständige Daten vorlagen

*ASD* atrialer Septumdefekt, *AVSD* atrioventrikulärer Septumdefekt, *DORV* double-outlet right ventricle, EMAH erwachsene Patientinnen mit angeborenen Herzfehlern,* NYHA* New York Heart Association, *PDA* persistierender Ductus arteriosus, *PFO* persistentes Foramen ovale, *TGA* Transposition der großen Arterien, *TOF* Fallot-Tetralogie, *UVH* univentrikuläres Herz, *VSD* ventrikulärer Septumdefekt

### Art der Entbindung

In der EMAH-Gruppe entbanden 42 Patientinnen (34,4 %) vaginal, während 79 Patientinnen (64,8 %) per Kaiserschnitt entbanden. Im übrigen Fall wurde das erste Zwillingskind vaginal und das zweite per Kaiserschnitt entbunden.

Alle Patientinnen der Vergleichsgruppe unterzogen sich einem Kaiserschnitt. In 19 Fällen kam es zu Mehrlingsgeburten (EMAH: *n* = 5 [4,1 %], VG: *n* = 14 [5,7 %]). Eine Patientin der Vergleichsgruppe gebar Drillinge.

In 49 Fällen (40,2 %) wurde ein Kaiserschnitt aus geburtshilflichen Gründen durchgeführt. In 24 Fällen (19,7 %) war die Indikation durch das Vitium bedingt, in zwei Fällen (1,6 %) aufgrund anderer Vorerkrankungen und in vier Fällen (3,3 %) gemischt (Herzfehler und geburtshilflich bedingt).

Bei Patientinnen mit milder kongenitaler Herzerkrankung erfolgte in 72,2 % (39 von 54) der Fälle ein Kaiserschnitt, im Vergleich zu 50,0 % (27 von 54 Patientinnen) bei moderatem und 85,7 % (12 von 14 Patientinnen) bei schwerem Vitium. Der Anteil der Kaiserschnitte unterschied sich nicht signifikant zwischen Patientinnen mit milder und jenen mit moderater oder schwerer kongenitaler Herzerkrankung (*p* = 1,0). Wurden jedoch Patientinnen mit milder und moderater Herzerkrankung zusammengefasst und mit jenen mit einem schweren Vitium verglichen, zeigte sich ein deutlicherer Unterschied (mild oder moderat: *n* = 67 [62 %], schwer: *n* = 12 [85,7 %]; *p* = 0,13).

### Qualifikation des Anästhesisten

Alle Patientinnen in den beiden höchsten Risikokategorien (*n* = 14, mWHO III und mWHO IV) wurden präoperativ in einem interdisziplinären Team aus Anästhesisten, Gynäkologen und EMAH-Spezialisten (Kinderkardiologie) besprochen. In der EMAH-Gruppe erfolgte bei 35,2 % (*n* = 43) der Patientinnen eine Konsultation durch einen Kardioanästhesisten. Bei Patientinnen der Klasse mWHO III und IV erfolgte in 10 Fällen die Betreuung durch Anästhesisten mit Erfahrung in der Kardio- oder Kinderkardioanästhesie, in zwei Fällen durch nicht spezialisierte Fachärzte für Anästhesie und in einem Fall durch eine Hebamme auf ausdrücklichen Wunsch der Patientin (Tab. 3).

**Tab. 3:** Überblick zum anästhesiologischen Management zur Entbindung von mWHO III und IV-Patienten. Eine Patientin wurde im Anschluss an einer Entbindung für eine geburtshilfliche Operation anästhesiologisch versorgt. Daher sind hier nur 13 der 14 Fälle mit mWHO III und IV dargestellt.

Art des Vitiums	mWHO-Klassifikation	Art der Entbindung	Art der Anästhesie	Monitoring	Narkoseinduktion	Narkoseaufrechterhaltung	Katecholamintherapie	Beatmung	Postoperative Therapie	Erfahrung des Anästhesisten
*Fontan-Zirkulation bei DILV *mit schwerer Aortenisthmusstenose	IV	Kaiserschnitt	AA mit ETI	Standard + Arterie	Etomidat	Inhalativ	Theodrenalin/Cafedrin	Standard PCV	Kinderkardiologische ITS	Kardioanästhesie
					Fentanyl					
					Succinylcholin					
*Hemifontan-Zirkulation bei TAT mit Pulmonal- und RPA-Stenose, TGA, Pulmonale Hypertension*	IV	Kaiserschnitt	AA mit ETI	Standard + Arterie + ZVK	Propofol	Inhalativ	Keine	Adaptiertes PCV (meanPIP < 8 cmH_2_O, low PEEP)	ITS	Kinderkardioanästhesie
					Fentanyl					
					Succinylcholin					
*Eingeschränkte Fontan-Zirkulation bei TAT*	IV	Kaiserschnitt	AA mit ETI	Standard + Arterie + ZVK	Thiopental	Inhalativ	Keine	Standard PCV	Kinderkardiologische ITS	Kardioanästhesie
					Fentanyl					
					Succinylcholin					
*Aorten- und Mitralstenose, pulmonale Hypertension*	IV	Kaiserschnitt	AA mit ETI	Standard + Arterie + ZVK	Etomidat	Inhalativ	Noradrenalin	Standard PCV	ITS	Kinderkardioanästhesie
					Fentanyl					
					Succinylcholin					
*HNOCM mit Endokardfibroelastose, ventrikuläre Tachykardie*	III	Kaiserschnitt	AA mit ETI	Standard + Arterie + ZVK	Etomidat	Inhalativ	Noradrenalin	Standard PCV	IMC	Kardioanästhesie
					Fentanyl					
					Succinylcholin					
*Fontan-Zirkulation bei univentrikulärem Herzen*	III	Kaiserschnitt	PDA	Standard	–	–	Phenylephrin	–	Kinderkardiologische ITS	Kardioanästhesie
*Fontan-Zirkulation bei univentrikulärem Herzen*	III	Geburt	Keine	Standard	–	–	Keine	–	Kreißsaal	Hebamme
*Schwere Pulmonalstenose nach DORV-Korrektur*	III	Kaiserschnitt	Spinalanästhesie	Standard	–	–	Theodrenalin/ Cafedrin	–	Kreißsaal	Facharzt
*Schwere Pulmonalstenose nach DORV-Korrektur*	III	Kaiserschnitt	Spinalanästhesie	Standard	–	–	Keine	–	IMC	Kardioanästhesie
*Korrigierte TGA mit pulmonaler Hypertension*	III	Kaiserschnitt	Spinalanästhesie	Standard	–	–	Keine	–	IMC	Kardioanästhesie
*Korrigierte TGA mit pulmonaler Hypertension*	III	Kaiserschnitt	Spinalanästhesie	Standard	–	–	Theodrenalin/Cafedrin	–	IMC	Kardioanästhesie
*Schwere Pulmonalstenose nach DORV-Korrektur mit TGA*	III	Kaiserschnitt	AA mit ETI	Standard + Arterie + ZVK	Propofol	Inhalativ	Keine	Adaptiertes PCV (meanPIP < 8 cmH_2_O, low PEEP)	IMC	Kardioanästhesie
					Fentanyl					
					Succinylcholin					
*Aorten- und Mitralstenose, Pulmonale Hypertension*	III	Kaiserschnitt	PDA	Standard	–	–	Keine	–	Normalstation mit Telemetrie	Facharzt

*AA* Allgemeinanästhesie, *DILV* double-inlet left ventricle, *DORV* double-outlet right ventricle, *HNOCM* hypertrophe nicht-obstruktive Kardiomyopathie, *ETI* endotracheale Intubation, *ITS* Intensivstation, *IMC* intermediate care, *meanPIP* mean peak inspiratory pressure, *PCV* pressure-controlled ventilation, *PDA* Periduralanästhesie, *PEEP* positive end-expiratory pressure, *RPA* rechte Pulmonalarterie, *TAT* Trikuspidalatresie, TGA Transposition der großen Arterien, *ZVK* zentraler Venenkatheter

### Anästhesiologisches Management

#### Anästhesie

In der EMAH-Gruppe wurde bei 88 Patientinnen (72,1 %) während der Entbindung eine Anästhesie durchgeführt, während 34 Patientinnen (27,9 %) ohne anästhesiologische Unterstützung entbanden.

Von den 79 Kaiserschnitten wurden 52 mit Spinalanästhesie, 11 mit Periduralanästhesie und 18 in Allgemeinanästhesie mit endotrachealer Intubation durchgeführt. In einem Fall wurde aufgrund des Verdachts einer unzureichenden Spinalanästhesie bei gleichzeitig hoher Dringlichkeit eine Allgemeinanästhesie durchgeführt. Ein weiterer Fall betraf den oben genannten Mischfall (vaginale Entbindung eines Zwillings, Sectio caesarea des zweiten Zwillings), sodass insgesamt 81 Anästhesieverfahren beschrieben sind. Von den 42 Patientinnen mit vaginaler Entbindung erhielten neun (21,4 %) eine Periduralanästhesie.

Die Art der Anästhesie war mit dem Schweregrad des angeborenen Herzfehlers assoziiert. In 10 der 18 Fälle (55,5 %), in denen der Kaiserschnitt in Allgemeinanästhesie durchgeführt wurde, beruhte die Indikation auf der kardialen Grunderkrankung oder einer damit verbundenen Antikoagulation. In fünf Fällen war eine geburtshilfliche Notfallsituation ausschlaggebend, in einem Fall eine nicht kardial bedingte Antikoagulation, und in zwei Fällen wurde der Grund nicht ausreichend detailliert dokumentiert. Insgesamt wurde die Allgemeinanästhesie bei EMAH-Patientinnen in 17,2 % (*n* = 21) der Fälle angewendet.

Bei allen geburtshilflichen Eingriffen (Kaiserschnitt und geburtshilfliche Operationen) wurden Spinal- und Periduralanästhesien signifikant häufiger bei Patientinnen mit niedrigeren mWHO-Klassen angewendet (*p* = 0,017). Bei Patientinnen mit mWHO-Klasse I oder II wurde in fünf Fällen (9,1 %) eine Periduralanästhesie, in 41 Fällen (74,5 %) eine Spinalanästhesie und in neun Fällen (16,4 %) eine Allgemeinanästhesie durchgeführt. Im Gegensatz dazu erhielten Patientinnen mit mWHO-Klasse III oder höher 22,6 % (*n* = 7) eine Periduralanästhesie, in 32,3 % (*n* = 10) ausschließlich eine Spinalanästhesie und in 38,7 % (*n* = 12) der Fälle eine Allgemeinanästhesie. Es wurden bei EMAH-Patientinnen weder Dosisanpassungen noch Änderungen des verwendeten Lokalanästhetikums vorgenommen.

#### Monitoring

Ein Standardmonitoring (bestehend aus EKG, Pulsoxymetrie und nicht-invasiver Blutdruckmessung) wurde in 70 Fällen (57,4 %) eingesetzt, während 26 Patientinnen (21,3 %) kein erweitertes Monitoring erhielten. Bei sieben Patientinnen wurde eine arterielle Blutdruckmessung etabliert, bei 14 (11,5 %) ein zentralvenöser Katheter (ZVK) gelegt, und bei fünf (4,1 %) Patientinnen wurden beide Verfahren kombiniert angewendet. Die Anlage einer arteriellen Kanüle war nahezu ausschließlich auf Patientinnen der mWHO-Klassen III (*n* = 2) und IV (*n* = 4) beschränkt (*p* < 0,001; eine Patientin in mWHO-Klasse II-III; Tab. 3). Im Gegensatz dazu wurden ZVK über alle mWHO-Kategorien hinweg eingesetzt (*p* = 0,176). Alle Patientinnen ohne Monitoring entbanden vaginal.

Eine Übersicht über das Management der Patientinnen mit mWHO-Klassen III und IV ist in Tab. 3 dargestellt.

### Postoperatives Management

EMAH-Patientinnen benötigten signifikant häufiger eine intensivmedizinische Überwachung nach der Entbindung als Patientinnen ohne angeborenen Herzfehler (ITS: EMAH *n* = 8 [6,6 %] vs. VG *n* = 4 [1,6 %], *p* = 0,02; IMC: EMAH *n* = 17 [13,9 %] vs. VG *n* = 9 [3,7 %], *p* < 0,001).

Während alle EMAH-Patientinnen der mWHO-Risikoklasse IV auf die ITS verlegt wurden, erhielten 77,8 % der Patientinnen mit mWHO-Klasse III eine erweiterte postoperative Überwachung (ITS: *n* = 1 [11,1 %]; IMC: *n* = 5 [55,6 %]; Telemetrie: *n* = 1 [11,1 %]). Fünf EMAH-Patientinnen (4,1 %) waren zunächst für eine erweiterte postoperative Überwachung vorgesehen, konnten jedoch noch am selben Tag aus dem Aufwachraum entlassen werden.

Die meisten EMAH-Patientinnen mit ITS- oder IMC-Indikation wurden am ersten (*n* = 19 [76 %]) oder zweiten (*n* = 4 [16 %]) postoperativen Tag auf die Normalstation verlegt. Eine EMAH-Patientin wurde erst am dritten postoperativen Tag aufgrund eines Blutungsereignisses verlegt, während bei einer Patientin der Vergleichsgruppe sich die Verlegung aufgrund einer Lungenembolie bis zum sechsten postoperativen Tag verzögerte.

Alle Patientinnen wurden interdisziplinär durch Teams aus Anästhesiologie, Intensivmedizin und Geburtshilfe betreut, wobei bei Bedarf eine kinderkardiologische Konsultation erfolgte.

Eine spezifische kardiale Medikation war bei neun EMAH-Patientinnen (7,4 %) erforderlich: β‑Blocker bei sieben (5,7 %), Diuretika und Antihypertensiva jeweils bei zwei (1,6 %) Patientinnen. Keine Patientin entwickelte im postoperativen Verlauf eine akute Herzinsuffizienz oder Arrhythmie. In einem Fall mit pulmonaler Hypertonie wurde eine Vasopressin-Infusion nach der Aufnahme auf die Intensivstation fortgesetzt und innerhalb von 12 Stunden beendet. Keine weitere EMAH-Patientin benötigte eine postoperative Katecholamintherapie.

Die postoperative Antikoagulation bei EMAH-Patientinnen der mWHO-Klasse IV erfolgte in drei von vier Fällen (75 %) mit intravenösem Heparin, während in einem Fall die Antikoagulation für 24 h pausiert wurde. In den übrigen Fällen mit intensivmedizinischer Überwachung wurde die Antikoagulation mit niedermolekularem Heparin durchgeführt oder vorübergehend für 24 Stunden ausgesetzt (Dalteparin: *n* = 4 [19,0 %]; Certoparin: *n* = 11 [52,4 %]; Enoxaparin: *n* = 2 [9,5 %]; keine Antikoagulation: *n* = 4 [19,0 %]).

Blutungsereignisse traten bei zwei von 13 EMAH-Patientinnen der mWHO-Klassen III und IV (15,4 %) sowie bei drei von 13 Patientinnen der Vergleichsgruppe mit ITS- oder IMC-Überwachung (23,1 %) auf.

### Outcome

In der EMAH-Gruppe traten keine maternalen Todesfälle auf, während in der Vergleichsgruppe eine Patientin durch Exsanguation verstarb, nachdem sie Blutprodukte abgelehnt hatte. Es wurden insgesamt sechs fetale Todesfälle dokumentiert (EMAH: *n* = 1 [0,8 %]; VG: *n* = 5 [2,0 %]) sowie drei Schwangerschaftsabbrüche (EMAH: *n* = 2 [1,6 %]; VG: *n* = 1 [0,4 %]). Insgesamt war die Rate anästhesieassoziierter Komplikationen in beiden Studiengruppen niedrig (4,9 % bei EMAH vs. 2,1 % bei VG; *p* = 0,191; Tab. 4).

**Tab. 4:** Übersicht über die aufgetretenen Komplikationen

Komplikationen	EMAH (*n* = 122)	VG (*n* = 244)	*p*-Wert
Geburtshilfe			
*Keine, n (%)*	82 (67,2)	145 (59,4)	0,171
*Geburtsstillstand, n (%)*	15 (12,3)	39 (15,9)	0,435
*Pathologisches CTG, n (%)*	16 (13,1)	47 (19,2)	0,186
*Blutung, n (%)*	14 (11,4)	15 (6,1)	0,099
*Andere geburtshilfliche Intervention, n (%)*	8 (6,5)	3 (1,2)	*0,008*
*Uterusruptur, n (%)*	1 (0,8)	9 (3,7)	0,175
*Andere, n (%)*	1 (0,8)	7 (2,7)	0,278
Anästhesie			
*Keine, n (%)*	116 (95,1)	239 (97,9)	0,191
*Arrhythmie, n (%)*	1 (0,8)	0	0,333
*Hypoxie, n (%)*	0	1 (0,4)	1,000
*Schwere Hypotension, n (%)*	4 (3,3)	3 (1,2)	1,000
*Andere, n (%)*	1 (0,8)	3 (1,2)	1,000

Statistische Unterschiede sind kursiv markiert (einzelne Patientinnen konnten mehr als ein Ereignis aufweisen). *p*-Werte werden zur deskriptiven Beschreibung von Gruppenunterschieden angegeben. Bei der Interpretation müssen die strukturellen Gruppenunterschiede hinsichtlich des Geburtsmodus (ausschließlich Sectio caesarea in der Vergleichsgruppe) beachtet werden. *CTG* Kardiotokogramm, *EMAH* erwachsene Patientinnen mit angeborenen Herzfehlern, *VG* Vergleichsgruppe

Der Bedarf an zusätzlichen geburtshilflichen Interventionen war in der EMAH-Gruppe signifikant höher (EMAH: *n* = 8 [6,6 %]; VG: *n* = 3 [1,2 %]; *p* = 0,008), wobei zu beachten ist, dass alle Patientinnen der Vergleichsgruppe eine Sectio caesarea erhielten. Blutungskomplikationen traten bei EMAH-Patientinnen häufiger auf, ohne jedoch eine statistische Signifikanz zu erreichen (*p* = 0,099; Tab. 4). Hinsichtlich anderer unerwünschter Ereignisse bestanden keine signifikanten Unterschiede zwischen den Studiengruppen (Tab. 4).

### Diskussion

Diese monozentrische Studie liefert drei zentrale Erkenntnisse zum anästhesiologischen Management von EMAH-Patientinnen im Kreißsaal:

1. *Sicherheit durch interdisziplinäre Versorgung: *Geburtshilfliche Anästhesie kann auch bei Patientinnen mit schweren kongenitalen Herzerkrankungen sicher durchgeführt werden, wenn die Betreuung interdisziplinär und durch erfahrene Anästhesisten erfolgt. Dies unterstreicht die entscheidende Bedeutung einer spezialisierten Ressourcenallokation.
2. *Erhöhte Rate an Kaiserschnitten:* EMAH-Patientinnen weisen eine höhere Rate an Kaiserschnittentbindungen als die Allgemeinbevölkerung auf [26]. Dies erfordert eine effiziente Nutzung anästhesiologischer Ressourcen sowie eine gezielte perioperative Planung.
3. *Erhöhte Prävalenz von Allgemeinanästhesie:* Allgemeinanästhesien werden bei EMAH-Patientinnen mit hohem perioperativem Risiko häufig durchgeführt, vor allem aufgrund einer bestehenden Antikoagulation oder der zugrunde liegenden kardialen Pathologie. Dies verdeutlicht die Notwendigkeit individualisierter anästhesiologischer Strategien.

Die zunehmende Prävalenz von EMAH ist in internationalen Registern, wie dem *European Registry of Pregnancy and Cardiac Disease* (ROPAC), gut dokumentiert. Diese liefern wertvolle Daten zu Morbidität und Mortalität bei Schwangeren mit unterschiedlichen Formen struktureller Herzerkrankungen. Ebenso bieten Risikostratifizierungsinstrumente wie der CARPREG-II- und der ZAHARA-Score wichtige Entscheidungshilfen für das Management dieser Patientinnengruppe [2, 20, 25]. Allerdings bieten diese Register nur begrenzte Handlungsempfehlungen für das konkrete anästhesiologische Management dieser Patientinnen in der klinischen Praxis.

Der Großteil der veröffentlichten Literatur zur geburtshilflichen Anästhesie bei EMAH-Patientinnen besteht aus Fallberichten und kleinen Fallserien [9, 11, 12, 28]. Selbst retrospektive monozentrische Analysen sind selten [10, 16]. Folglich wird ein Großteil des anästhesiologischen Wissens aus Daten zu kardiochirurgischen oder nicht-kardialen Operationen bei EMAH-Patientinnen oder aus kardioanästhesiologischen Studien zu nicht-kongenitalen Herzerkrankungen abgeleitet. Bis heute stellt das wissenschaftliche Statement der AHA die einzige umfassende Leitlinie zur anästhesiologischen Versorgung schwangerer Patientinnen mit kardialen Erkrankungen dar. Dies unterstreicht die anhaltende Relevanz von Erfahrungsberichten einzelner Zentren [5].

### Risikostratifizierung und anästhesiologisches Management

In unserem Patientenkollektiv wurden die meisten Patientinnen als niedrig bis moderat gefährdet eingestuft (mWHO I–III), wobei 20,5 % der intermediären Risikogruppe (mWHO II–III) und 11,5 % der Hochrisikogruppe (mWHO III–IV) zugeordnet wurden. Diese Verteilung entspricht den in anderen monozentrischen Studien berichteten Ergebnissen [10]. Obwohl die Kaiserschnittrate mit zunehmender Schwere des kongenitalen kardialen Vitiums anstieg, erreichte dieser Trend keine statistische Signifikanz, vermutlich aufgrund der insgesamt hohen Kaiserschnittrate in unserem Studienkollektiv [26]. Eine Registerstudie aus Norwegen von insgesamt 1.218.452 Entbindungen zeigte ebenso eine signifikant erhöhte Rate an Schnittentbindungen bei Patientinnen mit moderaten bis schweren Herzfehlern (26,2 %) [23]. Die hohe Rate an operativen Entbindungen unterstreicht die Notwendigkeit eines effizienten Ressourcenmanagements und einer spezialisierten Versorgungsplanung. Zwar ist der mütterliche Wunsch nach einer natürlichen Geburt nachvollziehbar, sollte aber insbesondere bei höhergradigen kongenitalen Herzfehlern interdisziplinär mit der Geburtshilfe und den (kinder-)kardiologischen EMAH-Spezialisten diskutiert werden, um das peripartale maternale Risiko zu minimieren. Die europäischen Leitlinien empfehlen einen Kaiserschnitt bei geburtshilflicher Indikation sowie bei bestimmten kardialen Hochrisikokonstellationen, insbesondere bei Patientinnen unter Vitamin-K-Antagonisten, bei Hochrisiko-Aortopathien, schwerer HCM mit LVOT-Obstruktion oder akuter Herzinsuffizienz [1]. Aus Sicht der Autoren muss dies auch bei Fontan-Patientinnen aufgrund der ungünstigen Effekte der Presswehen auf die Fontan-Physiologie diskutiert werden, wenn auch erfolgreiche Spontangeburten bei diesen Patientinnen beschrieben sind und solch ein Fall in dieser Studie erfasst wurde [28]. Die Indikation hierzu sollte ein interdisziplinäres und interprofessionelles „*Pregnancy Heart Team*“ stellen, welches bei allen Patientinnen der mWHO-Kategorie ≥ II-III konsultiert werden sollte [1]. Insbesondere bei Hochrisikopatienten sollten dabei die Backup-Strategien, wie z. B. die extrakorporale Membranoxygenierung (*extracorporeal cardiac life support*, ECLS), evaluiert werden. Hier sind entsprechend Experten wie etwa kongenitale Herzchirurgen und interventionelle Kardiologen zu konsultieren. Dies kann erfordern, dass solche Patientinnen im herzchirurgischen Operationssaal entbunden werden, was eine enge interdisziplinäre Absprache erforderlich macht.

In der EMAH-Gruppe kamen alle gängigen anästhesiologischen Verfahren zum Einsatz, wobei das Vorgehen bei Patientinnen mit niedrigem Risiko weitgehend dem routinemäßigen geburtshilflichen Standard entsprach. Allerdings war der Einsatz von Periduralanästhesie bei vaginalen Entbindungen etwas geringer als in der Allgemeinbevölkerung, was eher die lokale geburtshilfliche Praxis widerspiegelt als patientenindividuelle Kontraindikationen [3]. Neuraxiale Anästhesie, die von der AHA auch für Patientinnen der mWHO-Klasse III empfohlen wird, wurde in 46 % dieser Fälle angewendet, ein Anteil, der mit den 37,2 % in der Studie von Iluz-Freundlich et al. vergleichbar ist [5, 10]. Allgemeinanästhesien wurden bei höheren mWHO-Klassen generell häufiger und bei mWHO-IV-Patientinnen sogar in allen Fällen durchgeführt, was mit den Ergebnissen früherer Studien übereinstimmt [10, 16]. Diese Variabilität unterstreicht die Bedeutung individualisierter anästhesiologischer Konzepte, die Krankheitsausprägung, patientenindividuelle Faktoren und die Verfügbarkeit von Ressourcen sorgfältig in Einklang bringen [19, 20]. Dennoch ist zu betonen, dass die Periduralanästhesie insbesondere auch bei Patientinnen mit höhergradigen Vitien eine gute Option darstellen kann, wenn der Nutzen und die Risiken (z. B. Antikoagulation, Fontan-Physiologie) interdisziplinär im *Pregnancy Heart Team* diskutiert wurden. Zum einen kann sie den Geburtsschmerz und den damit verbundenen hämodynamischen Stress effektiv reduzieren, zum anderen kann sie im Falle eines Kaiserschnitts durch ein vorsichtiges Aufspritzen unter adäquatem Monitoring eine hohe hämodynamische Stabilität bieten. Daher wird diese Option auch explizit in den aktuellen europäischen Leitlinien für EMAH-Patientinnen mit hohem peripartalem Risiko empfohlen [1].

### Postoperatives Management

Postoperativ benötigten EMAH-Patientinnen mit 6,6 % Aufnahmen auf die Intensivstation und 13,9 % auf die IMC-Station (vs. 1,6 % bzw. 3,7 %) häufiger eine erweiterte Überwachung als die Vergleichsgruppe. Der Großteil der EMAH-Patientinnen mit erweitertem Überwachungsbedarf gehörte den mWHO-Klassen III und IV an, was die Komplexität ihrer kardialen Grunderkrankung widerspiegelt. Während nur fünf EMAH-Patientinnen, die ursprünglich für eine erweiterte Überwachung vorgesehen waren, noch am selben Tag aus dem Aufwachraum entlassen wurden, erfolgte bei der Mehrheit die Verlegung am ersten oder zweiten postoperativen Tag. Dies unterstreicht die Notwendigkeit einer verlängerten Überwachung bei Hochrisikopatientinnen selbst nach komplikationsloser Entbindung.

### Antikoagulation und Blutungsrisiko

Der hohe Anteil von Antikoagulanzientherapien bei EMAH-Patientinnen könnte die erhöhten Raten postpartaler Hämorrhagien sowie schwerer Blutungen in vergleichbaren Studien erklären. Art und Intensität der Antikoagulation stellen klinisch zentrale Einflussgrößen dar, da sie sowohl das Blutungsrisiko als auch die Wahl des Anästhesieverfahrens (neuraxial vs. Allgemeinanästhesie) und den Umfang des peri- und postoperativen Monitorings wesentlich beeinflussen. Da die präoperative antikoagulative Therapie aufgrund heterogener Indikationen und Therapieformen weder in das Matching einbezogen noch in den Analysen adjustiert oder stratifiziert werden konnte, ist ein relevanter Confounding-Effekt insbesondere für Blutungs- und Anästhesieendpunkte nicht auszuschließen. Dennoch unterstreichen diese Aspekte die klinische Relevanz der Antikoagulation als interdisziplinär zu berücksichtigenden Faktor in der Versorgung von EMAH-Patientinnen. In unserer Studie traten Blutungskomplikationen in der EMAH-Gruppe zwar häufiger auf als in der Vergleichsgruppe, allerdings war dieser Unterschied ohne statistische Signifikanz. Dieses Ergebnis ist jedoch vor dem Hintergrund struktureller Unterschiede zwischen den beiden Gruppen zu interpretieren. Die Vergleichsgruppe wies einen höheren Anteil multiparer Patientinnen auf, wobei eine höhere Parität einen bekannten unabhängigen Risikofaktor für postpartale Blutungen darstellt [17, 24]. Vor diesem Hintergrund ist nicht auszuschließen, dass das Blutungsrisiko der EMAH-Patientinnen im gruppenvergleichenden Ansatz unterschätzt wurde. Darüber hinaus können vorbestehende Gerinnungsstörungen in dieser Patientengruppe das Risiko für ein epidurales Hämatom mit entsprechenden neurologischen Komplikationen erhöhen [16].

### Fontan-Physiologie und individualisiertes Management

Geburtshilfliche Patientinnen mit Fontan-Physiologie sind eine besondere Herausforderung für die anästhesiologische Versorgung, die bisher überwiegend in Fallserien beschrieben wurde [28]. Diese Variabilität in der Versorgung dieser komplexen Patientinnen legt nahe, dass individualisierte Vorgehensweisen, die auf klinischer Erfahrung und patientenindividuellen Faktoren basieren, entscheidend für ein optimales Behandlungsergebnis sind.

Beispielsweise unterzog sich eine Patientin mit Aorten- und Mitralklappenstenose sowie sekundärer pulmonaler Hypertonie (initial mWHO III) zwei Kaiserschnitten: Der erste Eingriff erfolgte unter Periduralanästhesie, der zweite nach klinischer Verschlechterung auf mWHO IV unter Allgemeinanästhesie mit invasivem Monitoring und vasoaktiver Unterstützung. Dieser Fall verdeutlicht die dynamische Natur von kongenitalen Herzfehlern in der Schwangerschaft und die Notwendigkeit einer kontinuierlichen Reevaluation sowie einer interdisziplinären Zusammenarbeit, die auch die postoperative Phase einschließt [5, 14].

### Anästhesiologische Entscheidungsfindung

Alle anästhesiologischen Entscheidungen basierten auf einer umfassenden, multidisziplinären Beurteilung unter Einbeziehung der mWHO-Klasse, der Ventrikelfunktion, des Vorliegens einer Zyanose, bestehender Antikoagulation, Arrhythmien sowie der Patientenpräferenzen. Dadurch konnten medizinische, chirurgische, geburtshilfliche und psychosoziale Aspekte gleichermaßen berücksichtigt werden.

Die Wahl des Einleitungsanästhetikums richtete sich nach der hämodynamischen Stabilität. Bei Patientinnen mit ausgeprägten stenosierenden Vitien, pulmonaler Hypertonie oder Kardiomyopathie war Etomidat das Einleitungsmedikament der ersten Wahl. Aufgrund seines hämodynamisch neutralen Profils ermöglicht es eine rasche und sichere Atemwegssicherung – ein entscheidender Faktor bei Patientinnen mit fixiertem Herzzeitvolumen oder erhöhter rechtsventrikulärer Nachlast. Durch die Aufrechterhaltung des systemischen Gefäßwiderstands und der Myokardkontraktilität reduziert Etomidat das Risiko einer akuten Dekompensation während der Laryngoskopie und Intubation erheblich und ist damit insbesondere in Hochrisikosituationen (z. B. mWHO IV) besonders geeignet, auch wenn eine potenzielle vorübergehende Nebennierensuppression theoretisch berücksichtigt werden sollte [4, 13].

Bei Patientinnen mit Fontan- oder Hemi-Fontan-Physiologie wurden Propofol oder Thiopental in Einzelfällen als Alternativen eingesetzt, abhängig vom individuellen hämodynamischen Profil, etwa der Vorlastabhängigkeit oder der cavopulmonalen Shunt-Dynamik. Aufgrund ihrer vasodilatatorischen und negativ inotropen Effekte war jedoch eine besonders vorsichtige Titration erforderlich. Zudem erfolgte bei allen Patientinnen der mWHO-Klasse IV standardmäßig eine präoperative Anlage eines invasiven arteriellen Monitorings, um bei hämodynamischer Instabilität umgehend intervenieren zu können.

Bei Patientinnen unter therapeutischer Antikoagulation wurde bevorzugt eine Allgemeinanästhesie durchgeführt, um das Risiko eines neuraxialen Hämatoms zu minimieren. Wo immer möglich, kam jedoch eine Periduralanästhesie zum Einsatz, da sie eine überlegene hämodynamische Stabilität bietet. Auch Angst und individuelle Präferenzen spielten eine wesentliche Rolle bei der finalen Entscheidungsfindung, was die Bedeutung eines partizipativen Entscheidungsprozesses in dieser komplexen Patientengruppe unterstreicht. Obwohl das anästhesiologische Vorgehen konsequent auf einer individualisierten Nutzen-Risiko-Abwägung beruhte, schränkte der retrospektive Charakter dieser Studie eine systematische Auswertung der Entscheidungsprozesse ein.

### Limitationen

Diese Studie weist mehrere Limitationen auf: Erstens ist die Generalisierbarkeit der Ergebnisse eingeschränkt, da die Untersuchung an einem EMAH-Referenzzentrum mit spezialisierter kardiologischer und anästhesiologischer Expertise durchgeführt wurde. Dennoch verdeutlichen die Daten, was unter optimalen interdisziplinären Bedingungen erreicht werden kann, und können als Benchmark für Best-Practice-Modelle dienen. Auch nicht-tertiäre Zentren können von strukturierten Kooperationen oder Telekonsilen mit EMAH-Experten profitieren. Zweitens können aufgrund des retrospektiven Studiendesigns keine Kausalzusammenhänge nachgewiesen werden. Zudem erschwert die Heterogenität der kongenitalen kardialen Vitien eine direkte Vergleichbarkeit mit Patientinnen ohne angeborenen Herzfehler. Das Matching-Verfahren zielte darauf ab, zentrale Confounder wie Alter, BMI und schwangerschaftsassoziierte Begleiterkrankungen auszugleichen, konnte jedoch nicht alle Gruppenunterschiede eliminieren. Daher sollte der Vergleich deskriptiv interpretiert werden, um Komplikationsraten einzuordnen, und nicht, um Kausalitäten abzuleiten. Drittens umfasste die Vergleichsgruppe ausschließlich Kaiserschnitte. Vaginale Entbindungen wurden aufgrund uneinheitlicher Dokumentation im 20-jährigen Studienzeitraum nicht verwendet. Dies kann die Vergleichbarkeit der Gesamtergebnisse einschränken. Schwere Komplikationen (wie in dieser Studie definiert) wurden jedoch durchgehend durch Anästhesisten dokumentiert. Viertens stellt der lange Beobachtungszeitraum eine Limitation dar, da sich anästhesiologische Techniken über die Jahre potenziell verändert haben. In der anästhesiologischen Abteilung für Gynäkologie und Geburtshilfe führte jedoch eine hohe personelle Kontinuität nur zu geringfügigen Modifikationen der Anästhesieregime. Ein Nachteil dieser Stabilität ist, dass alternative Verfahren wie die kombinierte Spinal- und Periduralanästhesie nicht oder nur selten angewendet wurden. Fünftens könnte der deutliche Anstieg an EMAH-Schwangerschaften nach 2016 Veränderungen im Zuweisungsverhalten oder institutionellen Praktiken widerspiegeln, obwohl während des Studienzeitraums keine größeren personellen oder strukturellen Änderungen erfolgten. Dies war vermutlich zufällig bedingt, da zuvor mehr Patientinnen ihre Entbindung in heimatnahen Kliniken planten. Eine verstärkte Aufklärung in der EMAH-Ambulanz zugunsten einer Entbindung in unserem Zentrum könnte ebenfalls zu höheren Fallzahlen beigetragen haben. Zudem fiel dieser Zeitraum mit der Flüchtlingskrise in Europa zusammen, die möglicherweise die Inzidenz von angeborenen Herzfehlern im Kreißsaal beeinflusst hat. Zuletzt war die Dokumentation in den frühen 2000er-Jahren weniger detailliert. Wesentliche Basisinformationen standen jedoch durchgängig über das bereits seit 1996 implementierte elektronische Anästhesiedokumentationssystem zur Verfügung.

## Zusammenfassung

Bei EMAH-Patientinnen war auch bei schwerer Erkrankung in einem interdisziplinären Setting durch erfahrene Behandler eine sichere geburtshilfliche Anästhesie mit niedriger maternaler Komplikationsrate sowie ohne maternale Todesfälle oder schwere kardiale bzw. hämodynamische Ereignisse durchführbar.

Allgemeinanästhesien wurden bei EMAH-Patientinnen, die per Kaiserschnitt entbanden, häufiger angewendet, hauptsächlich aufgrund einer Antikoagulation oder hämodynamischen Überlegungen. Grundsätzlich waren alle Anästhesieverfahren praktikabel, sofern sie an das individuelle Risikoprofil und den klinischen Kontext angepasst wurden.

Unsere Ergebnisse bestätigen bestehende Empfehlungen, dass die Einbindung von Experten insbesondere bei Patientinnen mit schweren kongenitalen Vitien (mWHO III und IV) entscheidend ist. Strukturierte Versorgungsmodelle können anderen Krankenhäusern helfen, frühzeitig spezialisiertes Fachwissen einzubinden und damit die Versorgungsergebnisse zu optimieren. Wichtig ist zudem, dass schwangere EMAH-Patientinnen auch ungeplant als Notfälle in nicht spezialisierten Kliniken vorgestellt werden können. In diesen Situationen ist die frühe Erkennung des Bedarfs an einer spezialisierten Konsultation von zentraler Bedeutung. Die Übersetzung der mWHO-Kategorien in die Schweregrade mild, moderat oder schwer (im Einklang mit den AHA-Empfehlungen) erleichtert insbesondere in nicht spezialisierten Zentren die Identifikation von Patientinnen, die einer spezialisierten Versorgung bedürfen.

An unserer Einrichtung werden alle schwangeren EMAH-Patientinnen in der Kinderkardioanästhesie vorgestellt, um den individuellen Fall interdisziplinär zu besprechen. Bei Patientinnen der mWHO-Klassen III und IV erfolgt die Anästhesie durch Anästhesisten mit Expertise in der Kardio- oder Kinderkardioanästhesie. Dieses Modell gewährleistet einen gezielten Ressourceneinsatz und kann in anderen Zentren durch Telefon- oder Videokonsile adaptiert werden. Dabei stellen folgende Informationen wichtige Eckpfeiler für eine effektive Konsultation dar:

- zugrunde liegender Herzfehler und bisherige Behandlungen
- geplante Intervention und aktuelle kardiologische Befunde
- echokardiographische Ergebnisse (Pumpfunktion, Klappendysfunktion, pulmonale Hypertonie)
- Anamnese zu Arrhythmien, körperlicher Belastbarkeit, Dauermedikation und Gerinnungsstörungen
- weitere Begleiterkrankungen und frühere Schwangerschaften

Diese Studie zeigt, dass sorgfältige Planung, interdisziplinäre Zusammenarbeit und individuell angepasste anästhesiologische Strategien entscheidend für die Betreuung schwangerer EMAH-Patientinnen sind. Die Ergebnisse können als Modell für andere Zentren mit ähnlichen Rahmenbedingungen dienen und dazu beitragen, eine spezialisierte Versorgung zugänglich und effizient zu gestalten.

## Fazit für die Praxis

- Eine sichere geburtshilfliche Anästhesie ist auch bei EMAH-Patientinnen mit hoher Erkrankungsschwere möglich, wenn sie interdisziplinär und durch erfahrene Behandler erfolgt.
- Die Wahl des Anästhesieverfahrens sollte konsequent am individuellen Risikoprofil und klinischen Kontext orientiert werden.
- Allgemeinanästhesien kommen bei Hochrisikopatientinnen häufiger zum Einsatz, insbesondere bei bestehender Antikoagulation oder schweren hämodynamischen Einschränkungen.
- Bei Patientinnen mit mWHO III–IV sollte frühzeitig Expertise in der Kardio- oder Kinderkardioanästhesie eingebunden werden.
- Strukturierte interdisziplinäre Versorgungsmodelle erleichtern die Planung und verbessern die Versorgungsergebnisse.
- Auch in nicht spezialisierten Kliniken ist die frühzeitige Identifikation von Hochrisikopatientinnen und die Einleitung einer spezialisierten Konsultation entscheidend.
- Für eine effektive interdisziplinäre Abstimmung sind insbesondere Informationen zu Herzfehler, aktueller kardiologischer Befundlage, Echokardiographie, Rhythmusanamnese und Medikation essenziell.

## Data Availability

Die der Studie zugrunde liegenden Daten sind auf begründete Anfrage bei dem korrespondierenden Autor erhältlich.

## References

[1] De Backer J, Haugaa KH, Hasselberg NE et al (2025) 2025 ESC Guidelines for the management of cardiovascular disease and pregnancy. Eur Heart J 46:4462–4568. 10.1093/eurheartj/ehaf193 40878294 10.1093/eurheartj/ehaf193

[2] Balci A, Sollie-Szarynska KM, van der Bijl AGL et al (2014) Prospective validation and assessment of cardiovascular and offspring risk models for pregnant women with congenital heart disease. Heart 100:1373–1381. 10.1136/heartjnl-2014-305597 25034822 10.1136/heartjnl-2014-305597

[3] Bremerich DH, Greve S (2021) The new S1 guidelines “Obstetric analgesia and anesthesia”—Presentation and comments. Anaesthesist 70:229–236. 10.1007/s00101-020-00910-7 33464374 10.1007/s00101-020-00910-7

[4] Bruder EA, Ball IM, Ridi S et al (2015) Single induction dose of etomidate versus other induction agents for endotracheal intubation in critically ill patients. Cochrane Database of Systematic Reviews. 10.1002/14651858.CD010225.pub2 25568981 10.1002/14651858.CD010225.pub2 PMC6517008

[5] Canobbio MM, Warnes CA, Aboulhosn J et al (2017) Management of Pregnancy in Patients with Complex Congenital Heart Disease: A Scientific Statement for Healthcare Professionals from the American Heart Association. Circulation 135:e50–e87. 10.1161/CIR.0000000000000458 28082385 10.1161/CIR.0000000000000458

[6] Diller GP, Breithardt G, Baumgartner H (2011) Congenital Heart Defects in Adulthood. Deutsches Aerzteblatt Online. 10.3238/arztebl.2011.0452 10.3238/arztebl.2011.0452 PMC3139408 21776319

[7] von Elm E, Altman DG, Egger M et al (2008) The Strengthening the Reporting of Observational Studies in Epidemiology (STROBE) statement: guidelines for reporting observational studies. J Clin Epidemiol 61:344–349. 10.1016/j.jclinepi.2007.11.008 18313558 10.1016/j.jclinepi.2007.11.008

[8] Faraoni D, Zurakowski D, Vo D et al (2016) Post-Operative Outcomes in Children With and Without Congenital Heart Disease Undergoing Noncardiac Surgery. J Am Coll Cardiol 67:793–801. 10.1016/j.jacc.2015.11.057 26892415 10.1016/j.jacc.2015.11.057

[9] Goto S, Suzuki Y, Kurokawa S, Nagasaka Y (2025) Anesthesia management for cesarean delivery in patients with an arterial switch operation: a single center case series (2015–2023). Int J Obstet Anesth 61:104299. 10.1016/j.ijoa.2024.104299 39827660 10.1016/j.ijoa.2024.104299

[10] Iluz-Freundlich D, Vikhorova Y, Azem K et al (2024) Peripartum anesthesia management and outcomes of patients with congenital heart disease: a single-center retrospective analysis (2009–2023). Int J Obstet Anesth. 10.1016/j.ijoa.2024.104241 39227290 10.1016/j.ijoa.2024.104241

[11] Jha AK, Gundagurti B, Jha N (2025) Anesthetic Management and outcomes of pregnant women with Ebstein’s anomaly: Prospective report. American Association of Nurse Anesthesiology Journal 93:39–43. 10.70278/AANAJ/.0000001005 10.70278/AANAJ/.0000001005 39945150

[12] Leong RWL, Chen J, Mathews AMV, Kothandan H (2023) Anaesthesia of a parturient with uncorrected pentalogy of Fallot undergoing caesarean section and postpartum sterilisation. BMJ Case Rep 16:e251598. 10.1136/bcr-2022-251598 37802594 10.1136/bcr-2022-251598 PMC10565142

[13] Liu H, Miao JK, Cai M et al (2023) Anesthetic drug concentrations and placental transfer rate in fetus between term and preterm infants, twins, and singletons. Front Pharmacol. 10.3389/fphar.2023.1213734 37719861 10.3389/fphar.2023.1213734 PMC10502316

[14] Mehta LS, Warnes CA, Bradley E et al (2020) Cardiovascular Considerations in Caring for Pregnant Patients: A Scientific Statement from the American Heart Association. Circulation 141:e884–e903. 10.1161/CIR.0000000000000772 32362133 10.1161/CIR.0000000000000772

[15] Meng ML, Arendt KW, Banayan JM et al (2023) Anesthetic Care of the Pregnant Patient with Cardiovascular Disease: A Scientific Statement from the American Heart Association. Circulation. 10.1161/CIR.0000000000001121 36780370 10.1161/CIR.0000000000001121

[16] Monteiro RS, Dob DP, Cauldwell MR, Gatzoulis MA (2016) Anaesthetic management of parturients with univentricular congenital heart disease and the Fontan operation. Int J Obstet Anesth 28:83–91. 10.1016/j.ijoa.2016.08.004 27726918 10.1016/j.ijoa.2016.08.004

[17] Muñoz M, Stensballe J, Ducloy-Bouthors AS et al (2019) Patient blood management in obstetrics: prevention and treatment of postpartum haemorrhage. A NATA consensus statement. Blood Transfus 17:112–136. 10.2450/2019.0245-18 30865585 10.2450/2019.0245-18 PMC6476742

[18] Nasr VG, Markham LW, Clay M et al (2023) Perioperative Considerations for Pediatric Patients With Congenital Heart Disease Presenting for Noncardiac Procedures: A Scientific Statement From the American Heart Association. Circ Cardiovasc Qual Outcomes 16:E000113. 10.1161/HCQ.0000000000000113 36519439 10.1161/HCQ.0000000000000113

[19] Nicolson S (2024) Pediatric Cardiac Anesthesiologists: An Endangered Species. A Call to Action. World J Pediatr Congenit Heart Surg 15:139–141. 10.1177/21501351231214756 38018084 10.1177/21501351231214756

[20] Ramlakhan KP, Johnson MR, Lelonek M et al (2021) Congenital heart disease in the ESC EORP Registry of Pregnancy and Cardiac disease (ROPAC). International Journal of Cardiology Congenital Heart Disease 3:100107. 10.1016/j.ijcchd.2021.100107

[21] Regitz-Zagrosek V, Roos-Hesselink JW, Bauersachs J et al (2018) 2018 ESC Guidelines for the management of cardiovascular diseases during pregnancy. Eur Heart J. 10.1093/eurheartj/ehy340 30165544 10.1093/eurheartj/ehy340

[22] Roos-Hesselink JW, Pelosi C, Brida M et al (2024) Surveillance of adults with congenital heart disease: Current guidelines and actual clinical practice. Int J Cardiol. 10.1016/j.ijcard.2024.132022 38636602 10.1016/j.ijcard.2024.132022

[23] Sandberg M, Fomina T, Macsali F et al (2025) Labor onset and delivery mode in women with congenital heart disease—A nationwide cohort study. Acta Obstet Gynecol Scand 104:666–675. 10.1111/aogs.15064 39878100 10.1111/aogs.15064 PMC11919723

[24] Schlembach D, Annecke T, Girard T et al (2023) Peripartum Haemorrhage, Diagnosis and Therapy. Guideline of the DGGG, OEGGG and SGGG (S2k, AWMF Registry No. 015-063, August 2022). Geburtshilfe Frauenheilkd 83:1446–1490. 10.1055/a-2073-9615 40235829 10.1055/a-2073-9615 PMC11998639

[25] Silversides CK, Grewal J, Mason J et al (2018) Pregnancy Outcomes in Women With Heart Disease: The CARPREG II Study. J Am Coll Cardiol 71:2419–2430. 10.1016/j.jacc.2018.02.076 29793631 10.1016/j.jacc.2018.02.076

[26] Statistisches Bundesamt (2025) Fast ein Drittel aller Geburten im Jahr 2023 durch Kaiserschnitt. https://www.destatis.de/DE/Presse/Pressemitteilungen/2025/05/PD25_N024_23.html

[27] Stout KK, Daniels CJ, Aboulhosn JA et al (2019) 2018 AHA/ACC Guideline for the Management of Adults With Congenital Heart Disease: A Report of the American College of Cardiology/American Heart Association Task Force on Clinical Practice Guidelines. Circulation 139:e698–e800. 10.1161/CIR.0000000000000603 30586767 10.1161/CIR.0000000000000603

[28] Tiouririne M, De Souza DG, Beers KT, Yemen TA (2015) Anesthetic Management of Parturients with a Fontan Circulation. Semin Cardiothorac Vasc Anesth 19:203–209. 10.1177/1089253214566887 25586083 10.1177/1089253214566887

[29] Türkmen O, Akar Inan S (2025) Navigating pregnancy with cardiovascular disease: pathophysiology, risk stratification, and maternal-fetal outcomes. Turk J Med Sci 55:24–42. 10.55730/1300-0144.5940 40104301 10.55730/1300-0144.5940 PMC11913499

